# Supplementary material for: Kinase KEY1 controls pyrenoid condensate size throughout the cell cycle by disrupting phase separation interactions
Source: Nat Cell Biol. 2026 Mar 17;28(4):725–38. doi: 10.1038/s41556-026-01908-w (PMC13086578; doi:10.1038/s41556-026-01908-w)
Supplement: Supplementary file 1 — Reporting Summary [file 41556_2026_1908_MOESM1_ESM.pdf]

nature portfolio

Corresponding author(s): Martin C. Jonikas

Last updated by author(s): 01/23/2026

Reporting Summary

Nature Portfolio wishes to improve the reproducibility of the work that we publish. This form provides structure for consistency and transparency in reporting. For further information on Nature Portfolio policies, see our Editorial Policies and the Editorial Policy Checklist.

Statistics

For all statistical analyses, confirm that the following items are present in the figure legend, table legend, main text, or Methods section.

n/a	Confirmed	
☐	☒	The exact sample size (*n*) for each experimental group/condition, given as a discrete number and unit of measurement
☐	☒	A statement on whether measurements were taken from distinct samples or whether the same sample was measured repeatedly
☐	☒	The statistical test(s) used AND whether they are one- or two-sided<br>*Only common tests should be described solely by name; describe more complex techniques in the Methods section.*
☒	☐	A description of all covariates tested
☒	☐	A description of any assumptions or corrections, such as tests of normality and adjustment for multiple comparisons
☐	☒	A full description of the statistical parameters including central tendency (e.g. means) or other basic estimates (e.g. regression coefficient) AND variation (e.g. standard deviation) or associated estimates of uncertainty (e.g. confidence intervals)
☐	☒	For null hypothesis testing, the test statistic (e.g. *F*, *t*, *r*) with confidence intervals, effect sizes, degrees of freedom and *P* value noted<br>*Give P values as exact values whenever suitable.*
☒	☐	For Bayesian analysis, information on the choice of priors and Markov chain Monte Carlo settings
☒	☐	For hierarchical and complex designs, identification of the appropriate level for tests and full reporting of outcomes
☒	☐	Estimates of effect sizes (e.g. Cohen's *d*, Pearson's *r*), indicating how they were calculated

Our web collection on statistics for biologists contains articles on many of the points above.

Software and code

Policy information about availability of computer code

Data collection	Microscopy data was collected using Zeiss Zen Blue, Nikon Elements, VisiView. and MicroManager. SPR data was collected Biacore Software.
Data analysis	Mass Spec data was analyzed using Proteome Discoverer 2.5 and Scaffold 5 software. Microscopy data was analyzed in FIJI and with custom MATLAB scripts available on GitHub (https://github.com/linnealemma/KEY1_He-Lemma-etal). SPR data was analyzed using Biacore Evaluation Insight Software. Simulations were performed in COMSOL (https://github.com/amcalv/2025-Simulations-Kinase-KEY1-paper).

For manuscripts utilizing custom algorithms or software that are central to the research but not yet described in published literature, software must be made available to editors and reviewers. We strongly encourage code deposition in a community repository (e.g. GitHub). See the Nature Portfolio guidelines for submitting code & software for further information.

Data

Policy information about availability of data

All manuscripts must include a data availability statement. This statement should provide the following information, where applicable:

- Accession codes, unique identifiers, or web links for publicly available datasets
- A description of any restrictions on data availability
- For clinical datasets or third party data, please ensure that the statement adheres to our policy

Data available in the supplemental information and additional supporting data is available on request from M.C. Jonikas.

Research involving human participants, their data, or biological material

Policy information about studies with human participants or human data. See also policy information about sex, gender (identity/presentation), and sexual orientation and race, ethnicity and racism.

Reporting on sex and gender	NA
Reporting on race, ethnicity, or other socially relevant groupings	NA
Population characteristics	NA
Recruitment	NA
Ethics oversight	NA

Note that full information on the approval of the study protocol must also be provided in the manuscript.

Field-specific reporting

Please select the one below that is the best fit for your research. If you are not sure, read the appropriate sections before making your selection.

☒ Life sciences ☐ Behavioural & social sciences ☐ Ecological, evolutionary & environmental sciences

For a reference copy of the document with all sections, see nature.com/documents/nr-reporting-summary-flat.pdf

Life sciences study design

All studies must disclose on these points even when the disclosure is negative.

Sample size

No statistical methods were used to pre-determine sample sizes. Sample sizes were determined by convention in the literature and quality of the data to distinguish wild-type behaviors from mutant cell behaviors.

Data exclusions

For live cell microscopy of fluorescently-tagged Chlamydomonas strains, cells were excluded from analysis if they were dead, as assessed by their autofluorescence at 560 nm excitation. Images were excluded from analysis if the offset between the fluorescent channels caused the outline of the cell to shift between colors.

Replication

All experiments were independently repeated as described below.

Co-immunoprecipitation mass spectrometry experiment was performed 2 times and is consistent with previous study (Mackinder et al., 2017; PMID: 28938113). (Fig. 1b-c)
TEMs were performed 2 times and results were consistent with fluorescence microscopy data. (Fig. 1d-e)
Imaging of wild-type, key1 mutants and KEY1 rescue pyrenoids was performed 3 times with similar results. (Fig.1f-h)
Spot test assay was performed 3 times with similar results. (Fig. 1k)
The confocal imaging of pyrenoid dynamics during cell division was performed independently 3 times (all EPYC1-Venus strains and key1-1;RBCS1-Venus;KEY1-SNAP) and 5 times (Wild type and key1-1 with RBCS1-Venus). Results between replicates were similar (Fig. 2a,b,d,e; Extended Data Fig. 2b,c,e,f,h,i).
Phos-tag gel-based western blot on cell lysate was performed 3 times with similar results (Fig. 3a).
Phos-tag gel-based western blot on cell lysate treated with KEY1 or phosphatase was performed 1 time and was consistent with Fig. 3a and Fig. 3c (Fig. 3b, Extended Data 5c).
The Coomassie Phos-tag gel for in vitro KEY1 activity on EPYC1 was performed 3 independent times. Results between replicates were similar (Fig. 3c).
Mass spec on in vivo EPYC1 was not replicated, but is consistent with previous studies (Wang et al., 2014; doi.:10.1074/mcp.M114.038281) (Fig. 3d).
Mass spec on in vitro EPYC1 protein was performed 2 independent times (Fig. 3e-g).
The diurnal Phos-tag gel-based western blot was performed 3 independent times with similar results (Fig. 3k).
The in vitro phase diagram was measured 3 times (Fig. 4a-d).
The FCS experiment to measure the binding between Rubisco and phosphorylated EPYC1 was performed 2 times (Fig. 4f).
The pyrenoid Phos-tag gel-based western blot was performed 3 independent times with similar results (Fig. 4g).
The SNAP labeling and confocal imaging of KEY1-SNAP and KEY1ΔRBM-SNAP were performed 3 independent times with n>50 cells for each strain in each replicate. Results between replicates were similar (Fig. 5a, Extended Data Fig. 8a-c).
SPR measurements of KEY1's Rubisco binding motif interaction with Rubisco was performed 8 times (Fig. 5d, Extended Data Fig. 7).
The Phos-tag gel-based western blot on KEY1-SNAP and KEY1ΔRBM-SNAP was performed 2 times with similar result (Fig. 5e).
RT-qPCR was performed 2 times (Extended Data Fig. 1i).
The time course of EPYC1-Venus was performed 4 independent times with n=10 cells for each experiment with similar results (Extended Data Fig. 3).
The confocal imaging to the phosphonull mutant with EPYC1-Venus was performed independently 3 times with similar results (Extended Data Fig. 4).
The Coomassie and anti-His western for KEY1 protein purifications was performed once at the time of purification. Purity was assessed two times later for E. coli-purified KEY1 through Coomassie stain with similar results (Extended Data Fig 5a,b).

The Coomassie Phos-tag gel of the samples sent for mass spec was performed 2 times, once for each mass spec replicate. The results between replicates were similar (Extended Data Fig. 5d).
Diurnal imaging of KEY1-Venus localization was performed once. Results were consistent with 3 independent measurements at t=9 hours, 3 independent measurements of mixed cell cycle, and KEY1-SNAP diurnal localization (Extended Data Fig. 6b).
The in vitro assay for KEY1-ΔRBM activity on EPYC1 was performed independently 3 times with similar results (Extended Data Fig. 8g).

Randomization: No randomization was used in this study.

Blinding: No blinding was used in this study.

Reporting for specific materials, systems and methods

We require information from authors about some types of materials, experimental systems and methods used in many studies. Here, indicate whether each material, system or method listed is relevant to your study. If you are not sure if a list item applies to your research, read the appropriate section before selecting a response.

Materials & experimental systems

n/a	Involved in the study	
☐	☒	Antibodies
☐	☒	Eukaryotic cell lines
☒	☐	Palaeontology and archaeology
☒	☐	Animals and other organisms
☒	☐	Clinical data
☒	☐	Dual use research of concern
☒	☐	Plants

Methods

n/a	Involved in the study	
☒	☐	ChIP-seq
☒	☐	Flow cytometry
☒	☐	MRI-based neuroimaging

Antibodies

Antibodies used: Rabbit IgG in Goat, polyclonal secondary HRP from Invitrogen Cat#: P131466. Rabbit polyclonal anti-EPYC1 from Mackinder et al., 2016.

Validation: Validated in previous study Mackinder et al., 2016.

Eukaryotic cell lines

Policy information about cell lines and Sex and Gender in Research

Cell line source(s): C. reinhardtii wild-type cells were CC-4453 , mating type (+).
C. reinhardtii key1 mutant cells were from CLiP mutant library. They can be found at the Chlamydomonas Resource Center: key1-1 LMJ.RY0402.107748 and key1-2 LMJ.RY0402.168929.
Remaining strains were generated for the study and are described in Supplementary Table 4. They are available through the Chlamydomonas Resource Center.

Authentication: key1 mutant cassette insertion sites were validated by PCR as described in the Methods.

Mycoplasma contamination: NA

Commonly misidentified lines (See ICLAC register): NA

Plants

Seed stocks: NA

Novel plant genotypes: NA

Authentication: NA
