# Supplementary material for: Kinase KEY1 controls pyrenoid condensate size throughout the cell cycle by disrupting phase separation interactions
Source: Nat Cell Biol. 2026 Mar 17;28(4):725–38. doi: 10.1038/s41556-026-01908-w (PMC13086578; doi:10.1038/s41556-026-01908-w)

Supplementary Figure of Full Gel Images

Kinase KEY1 controls pyrenoid condensate size throughout the cell cycle by disrupting phase separation interactions

Authors:
Shan He[1,2,3,11], Linnea M. Lemma[2,4,11], Alejandro Martinez-Calvo[5,6,7], Guanhua He[1], Jessica H. Hennacy[1], Lianyong Wang[1,8], Sabrina L. Ergun[1,2], Ashwani K. Rai[1], Colton Wang[1], Luke Bunday[1], Angelo Kayser-Browne[1], Quan Wang[9,10], Clifford P. Brangwynne[2,4,7], Ned S. Wingreen[1,5,9], Martin C. Jonikas[1,2,4]

[1]Department of Molecular Biology, Princeton University, Princeton, NJ 08544, USA
[2]Howard Hughes Medical Institute, Princeton University, Princeton, NJ 08544, USA
[3]Current address: Department of Botany, University of Wisconsin-Madison, Madison, WI 53706, USA
[4]Omenn-Darling Bioengineering Institute, Princeton University, Princeton, NJ 08544, USA
[5]Princeton Center for Theoretical Science, Princeton University, Princeton, NJ 08544, USA
[6]Department of Physics, Princeton University, Princeton, NJ 08544, USA
[7]Department of Chemical and Biological Engineering, Princeton University, Princeton, NJ 08544, USA
[8]Current address: Institute for Plant-Human Interface, Northeastern University, Boston, MA 02120, USA
[9]Lewis-Sigler Institute for Integrative Genomics, Princeton University, Princeton, NJ 08544, USA
[10]Current address: Laboratory of Chemical Physics, National Institute of Diabetes and Digestive and Kidney Diseases, National Institutes of Health, Bethesda, MD 20892, USA
[11]These authors contributed equally to this work: Shan He, Linnea Lemma

e-mail: mjonikas@princeton.edu, wingreen@princeton.edu

Related to Fig. 1k

Full images of spot test growth assay plates.

Very Low CO_2 (0.004%)
TP
200 uE

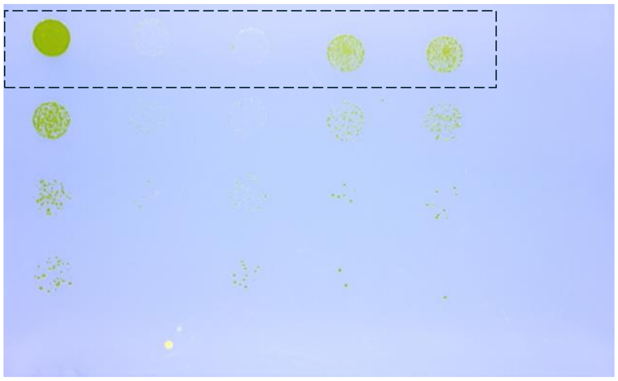

Air CO_2 (0.04%)
TP
200 uE

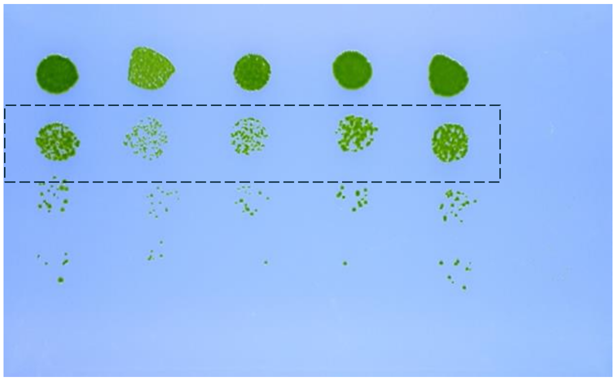

High CO_2 (3%)
TP
200 uE

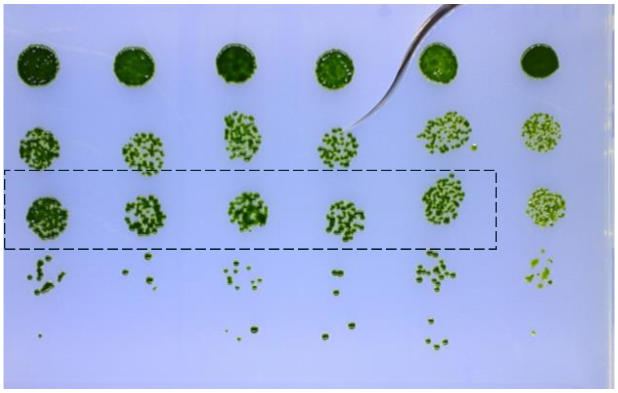

Air CO_2 (0.04%)
TAP
0 uE

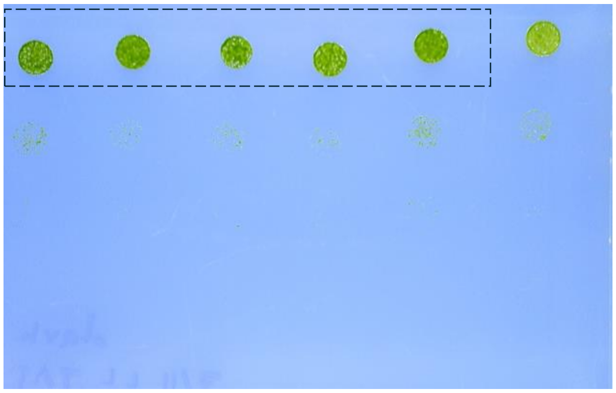

Related to Fig. 3a

Full image of phos-tag based western blot for anti-EPYC1 on cell lysates from wild-type, *key1* mutants and KEY1 rescue strains.

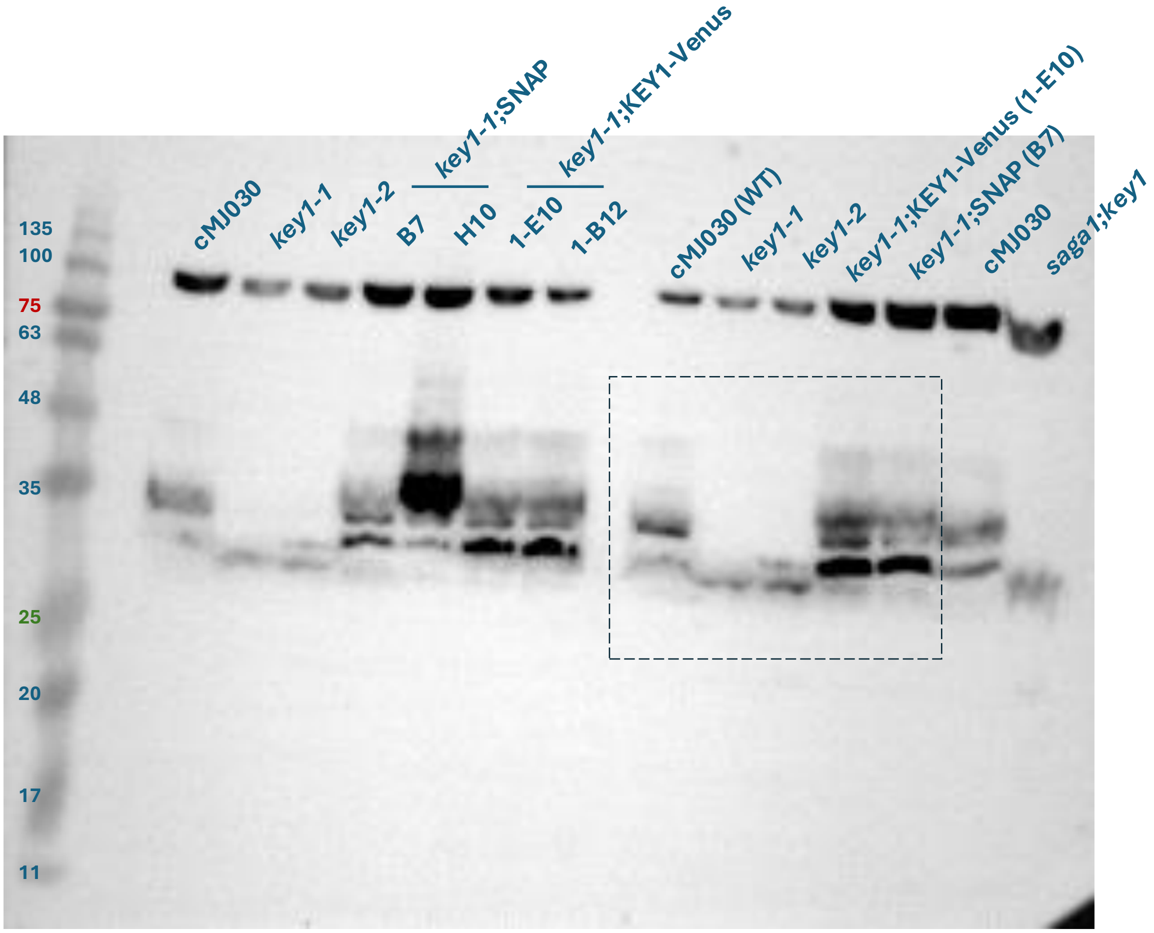

Related to Fig. 3b

Full image of phos-tag based western blot for anti-EPYC1 on cell lysates treated with KEY1 and lambda phosphatase.

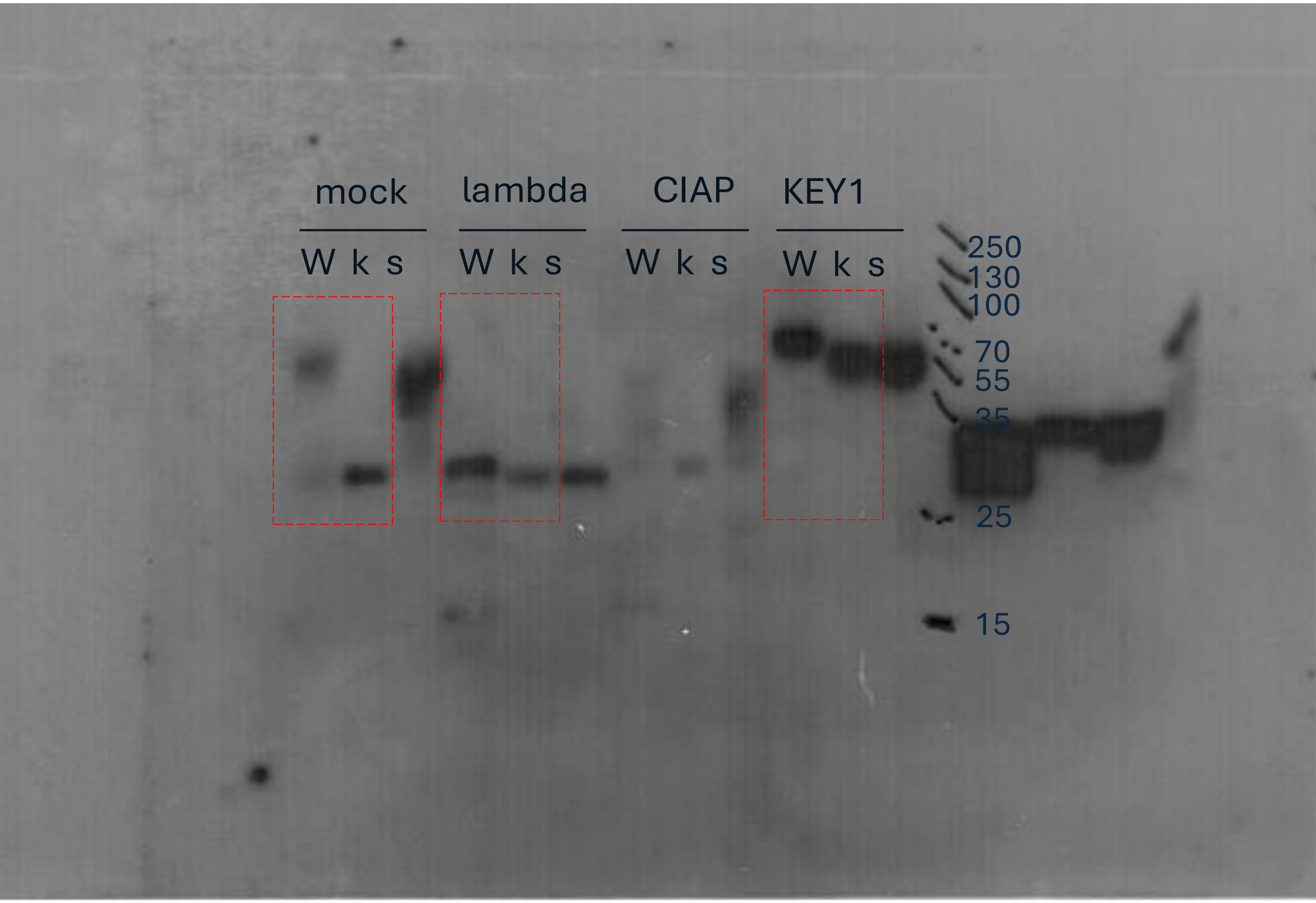

W: WT (cMJ030)
k: *key1-1*
s: *not relevant to this study*

Related to Fig. 3c

Full image of Coomassie-stained phos-tag gel of KEY1-treated EPYC1-gfp.

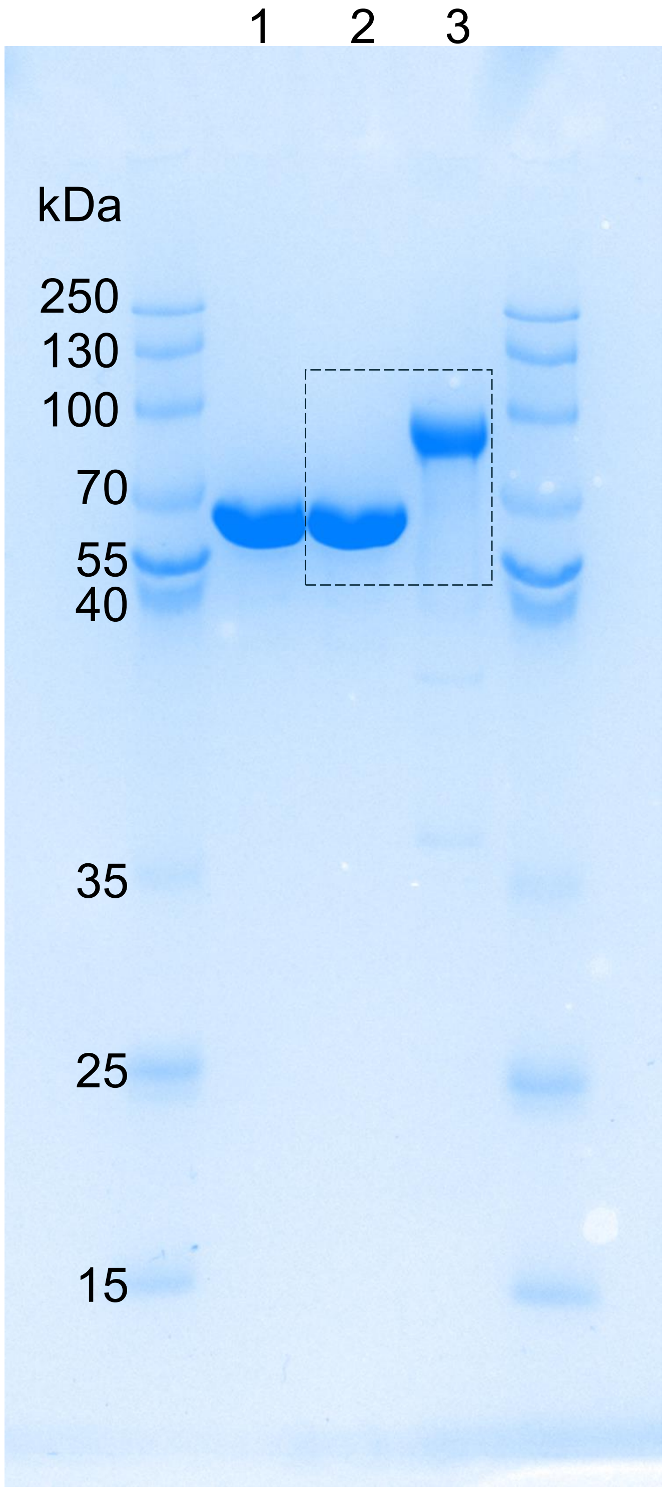

1 - 14 µM EPYC1-gfp untreated

2 - 14 µM EPYC1-gfp mock treated

3 -14 µM EPYC1-gfp + 3 µM KEY1

Related to Fig. 3k

Full image of phos-tag gel based western blot for anti-EPYC1 on wild-type cell lysate from synchronized cells across the cell cycle.

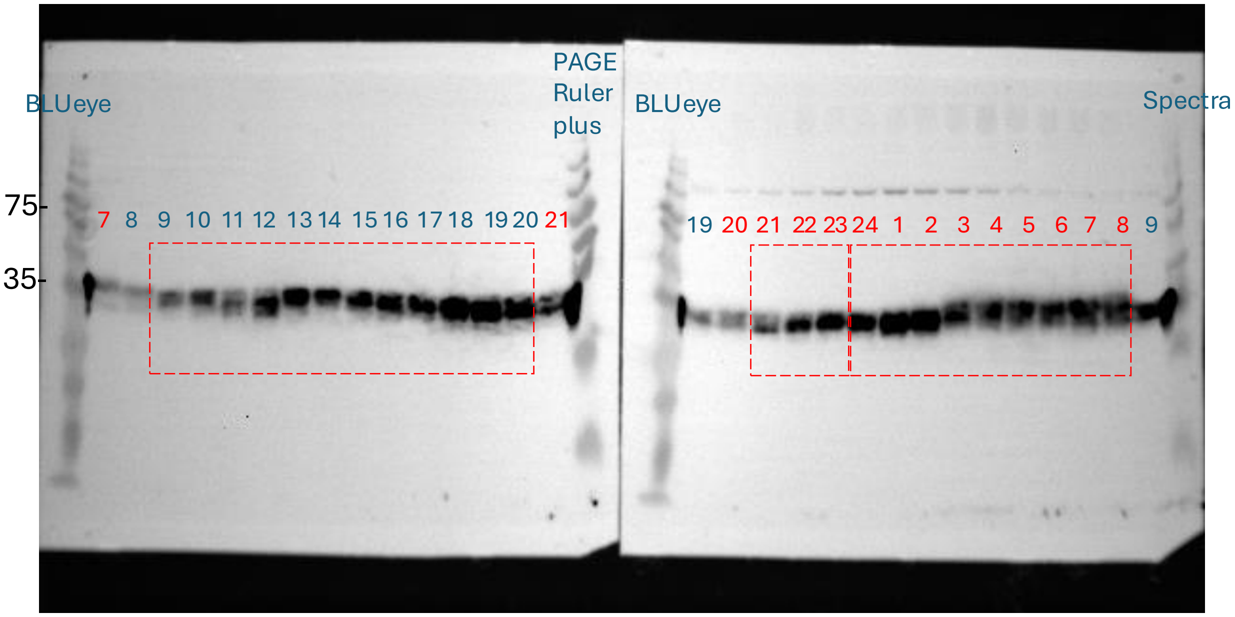

Related to Fig. 4g

Full images of SDS and phos-tag gel based western blot for EPYC1 on wild-type cell lysate.

SDS gel
Anti SAGA1 (binds EPYC1)

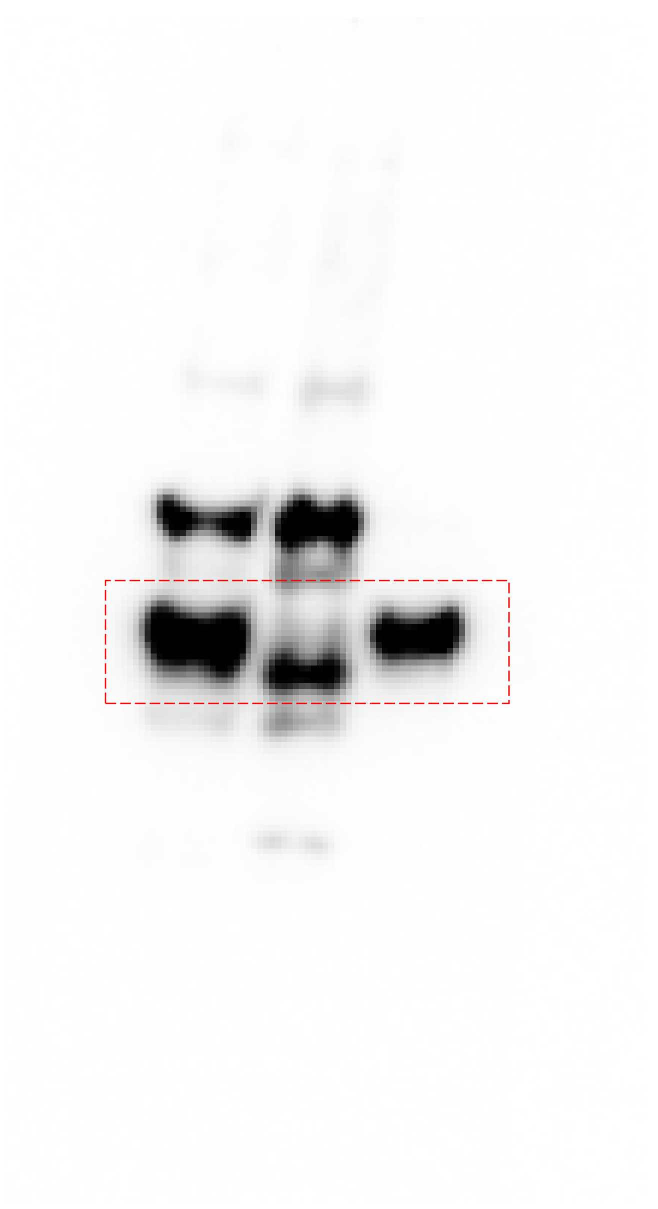

Phos-tag gel
Anti EPYC1

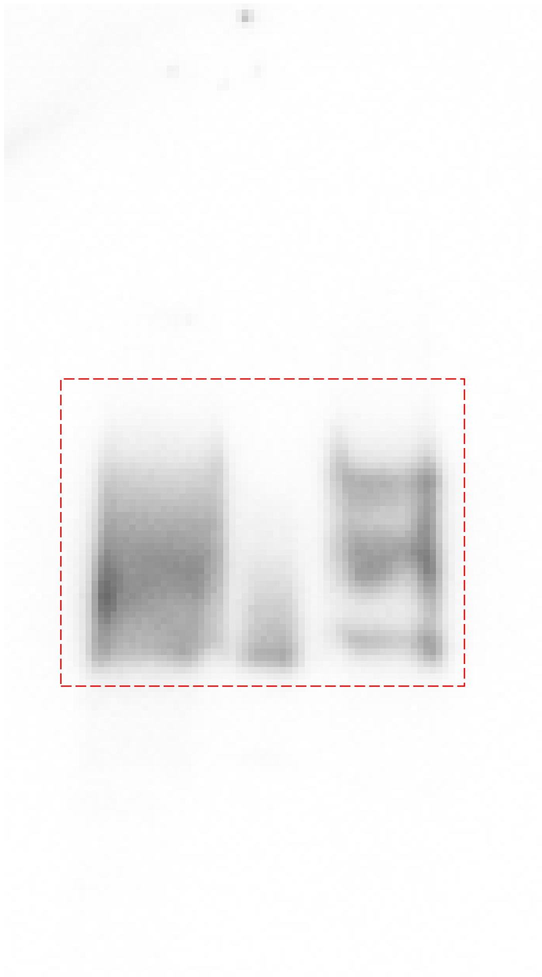

Related to Fig. 5e

Full image of phos-tag gel based western blot for anti-EPYC1 on RBCS1-Venus tagged *key1-1* mutants rescued by KEY1-SNAP or KEY1-ΔRBM.

2023_04_12_154358_Phos-tag_anti-EPYC1_2S

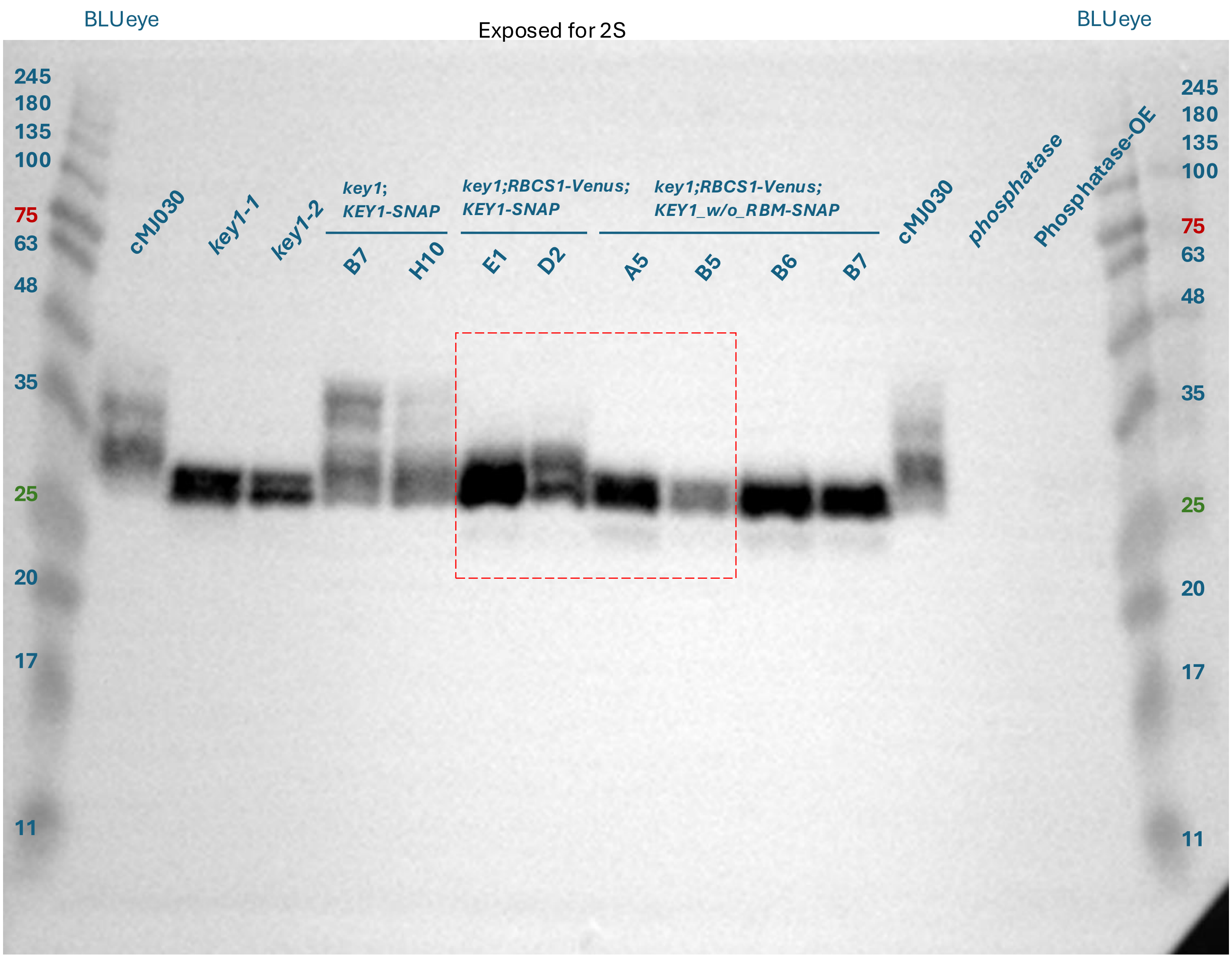

Related to Extended Data Fig. 1g

Full image of the agarose gel for characterizing *key1-2*.

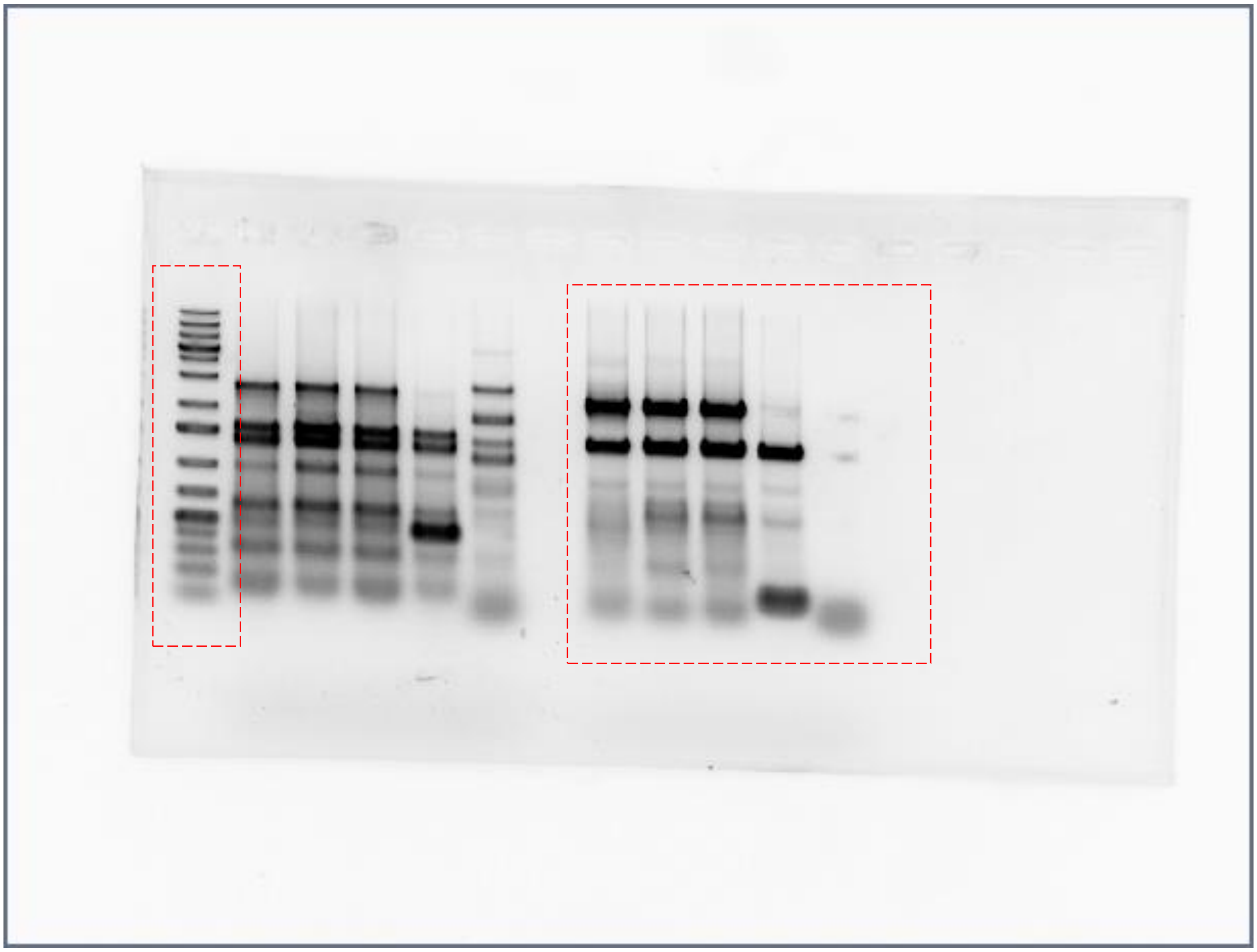

Related to Extended Data Fig. 1h

Full image of the agarose gel for characterizing *key1-2*.

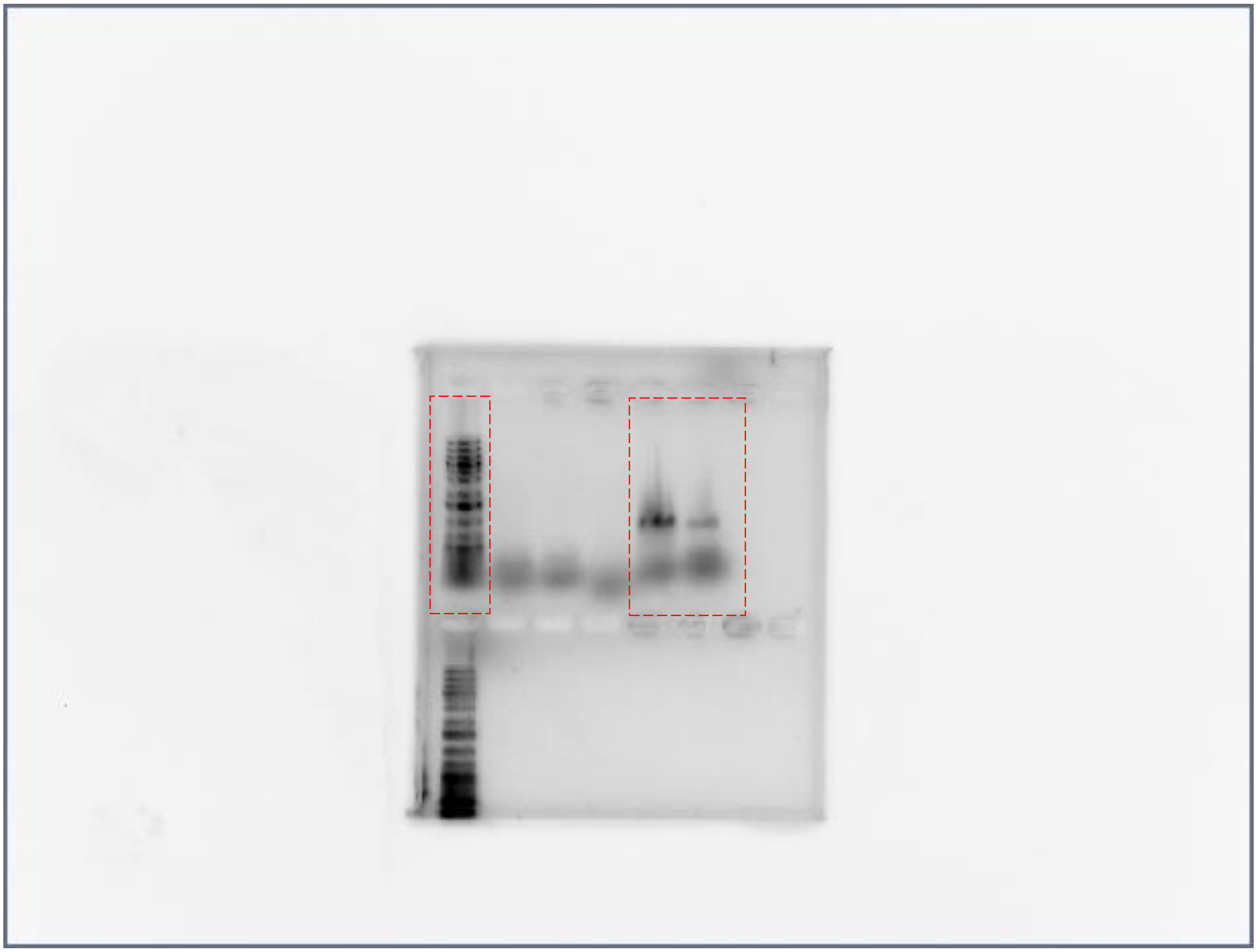

Related to Extended Data Fig. 5c

Full image of phos-tag gel based western blot for anti-EPYC1 of purified EPYC1 with or without Lambda phosphatase, Casein kinase II, *E. coli* expressed KEY1 or insect cell expressed KEY1.

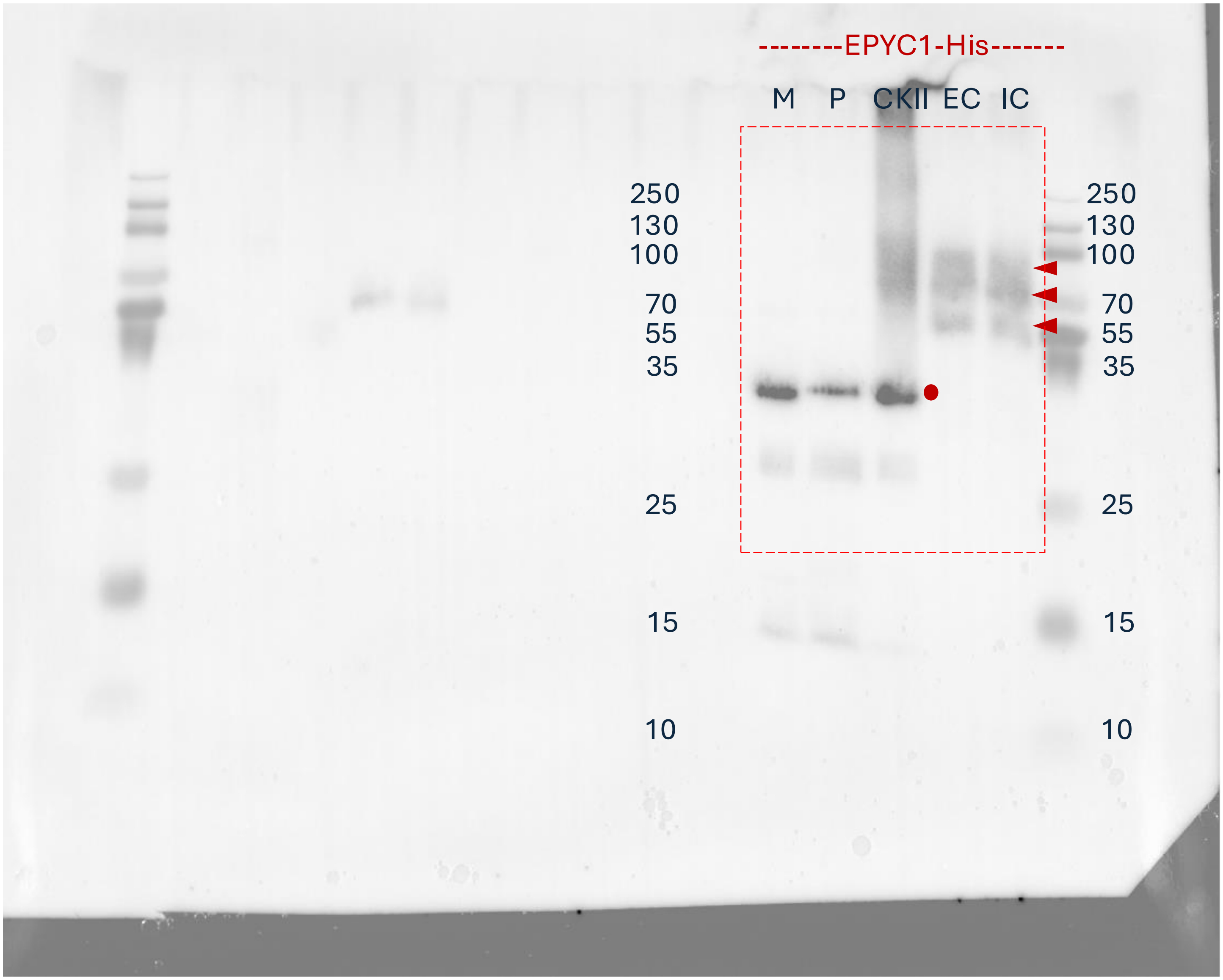

Related to Extended Data Fig. 5c

Full image of Coomassie stained phos-tag gel of E. coli expressed EPYC1-GFP samples prepared for phosphoproteomics.

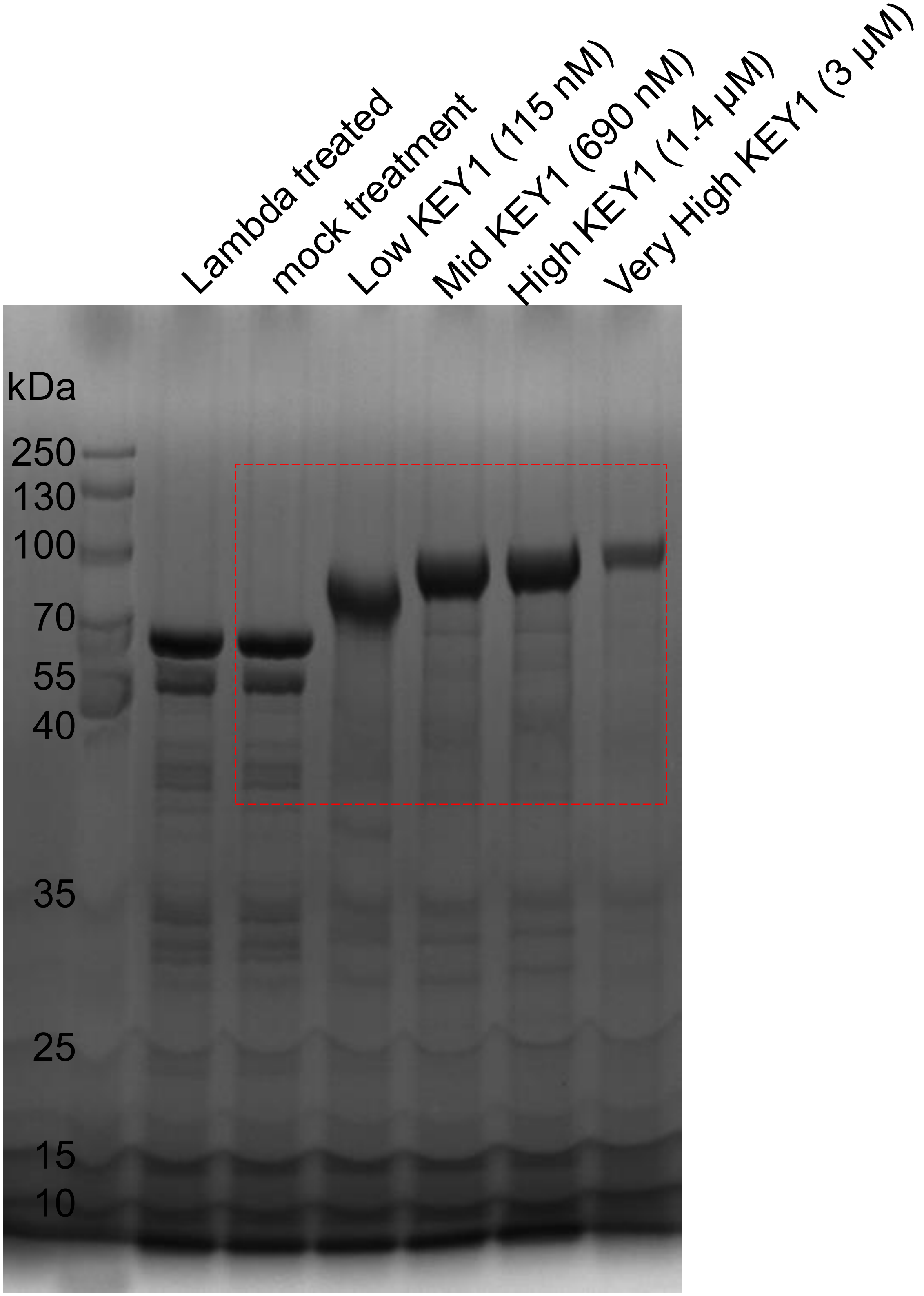

Related to Extended Data Fig. 8g

Full image of Coomassie-stained phos-tag gel of EPYC1-GFP treated with or without KEY1-WT or KEY1-ΔRBM.

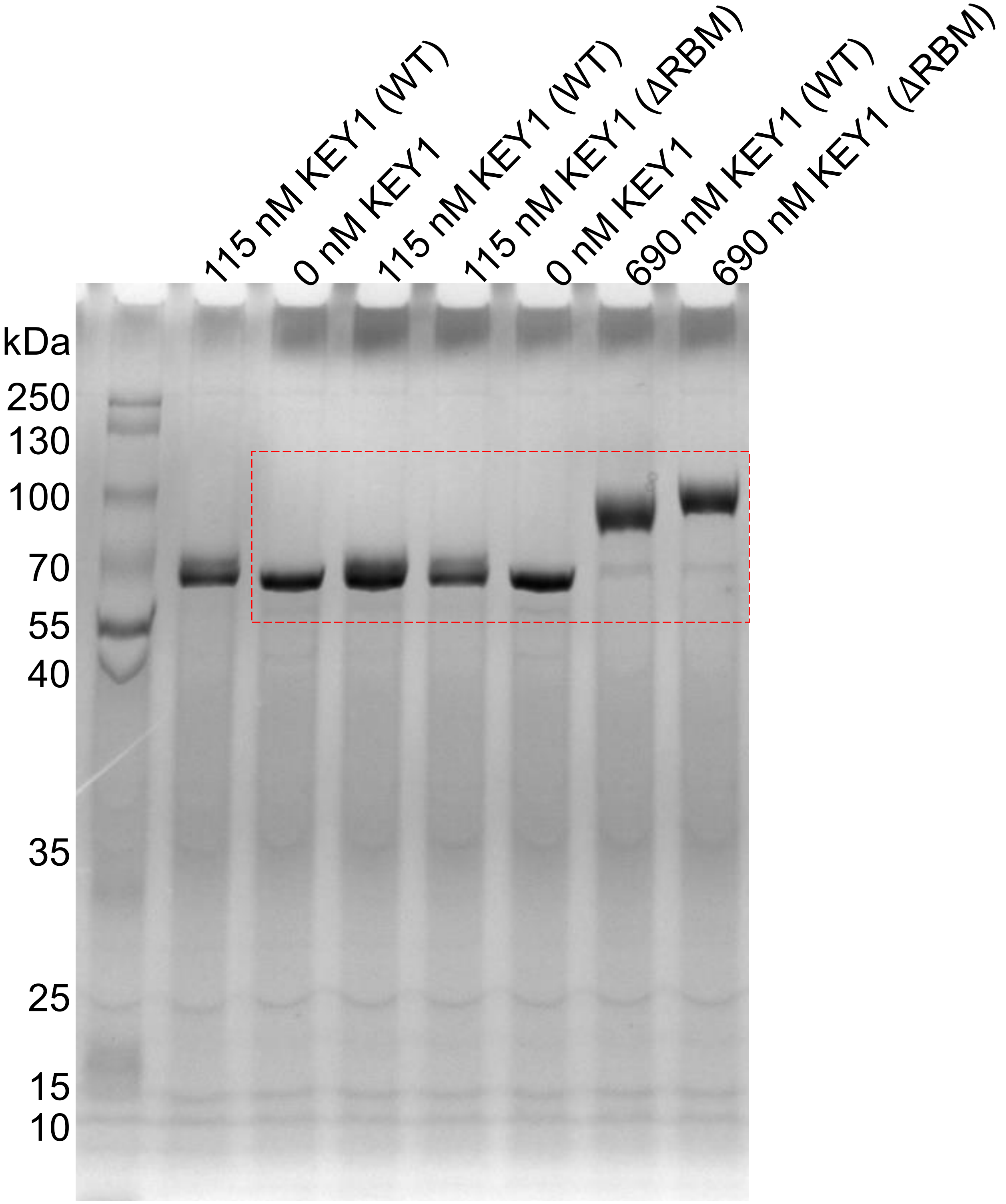

Supplement: Supplementary file 19 — Clearly labelled images of the unprocessed gels and blots used in the paper. [file 41556_2026_1908_MOESM19_ESM.pdf]
